# The scorpions of Yunnan (China): updated identification key, new record and redescriptions of *Euscorpiops kubani* and *E. shidian* (Arachnida, Scorpiones)

**DOI:** 10.3897/zookeys.82.715

**Published:** 2011-02-23

**Authors:** Zhiyong Di, Yawen He, Yingliang Wu, Zhijian Cao, Hui Liu, Dahe Jiang, Wenxin Li

**Affiliations:** College of Life Sciences, Wuhan University, Wuhan 430072, China

**Keywords:** Buthidae, Euscorpiidae, new record, taxonomy, redescriptions, Yunnan, China

## Abstract

We present an identification key to the scorpion species of Yunnan (China) with notes on the distribution and ecology. Euscorpiops kubani is recorded for the first time for China. The redescriptions of Euscorpiops shidian and Euscorpiops kubani are provided. The number of known scorpion species from Yunnan is raised to nine.

## Introduction

Yunnan province located in the junction of world’s two major biodiversity hotspots (21°8'32"–29°15'8"N, 97°31'39"–106°11'47"E), is the transition area from the high altitudes of Qinghai-Tibet plateau to low altitude peninsular Malaysia. Almost all of the terrestrial ecosystems can be found in Yunnan, including forests, shrubs, meadows, swamps and deserts (Chen et al. 2010). Because of the complex and varied terrains and landforms, the different areas can be divided into seven zones according to different climate types: North tropics, South Subtropical, Central Subtropical, North Subtropical, South Temperate, Central Temperate and Plateau climate zones.

The terrestrial diversity in Yunnan can meet the specific habitats demand of different species (Dong and Guo 2008). The scorpion biodiversity of the Yunnan is enormous compared to other provinces of China. Researchers erected six new species from the 1990s (Kovařík 1994, 2000; Qi et al. 2005; Di et al. 2010a, 2010b). The genus Euscorpiops Vachon, 1980 with its preference to humid habitats reaches its northern distribution limit in Yunnan, and the distribution of some species is restricted to this area.

There are nine species, belonging to two families: Buthidae: LychasC.L. Koch, 1845, Euscorpiidae: Euscorpiops and Scorpiops Peters, 1861have been recorded for this area. With seven species of Euscorpiops occur in Yunnan province more than a third of the total known species of this genus in the world (7/19); all them with similar coloration, morphology and close distribution.

## Material and methods

Illustrations and measurements were produced using a Motic K-700L stereomicroscope with a drawing device and an ocular micrometer. The photos were taken with an Olympus C7070 camera. Measurements follow Sissom (1990), and are given in mm. Trichobothrial notations follow Vachon (1974) and morphological terminology mostly follows Hjelle (1990). Terminology of metasomal carination follows Vachon (1952), Prendini (2000) and Soleglad and Sissom (2001) for pedipalp chela carinae. Specimens are deposited in the Museum of Wuhan University, Wuhan, China (MWHU), and Biological specimens Herbarium of Dali College, Yunnan, China (BHDC). Other abbreviations of collections: FKCP: private collection of F. Kovařík, Prague, Czech Republic; MHBU: Museum of the College of Life Sciences, Hebei University, Baoding, China; MNHN: Muséum National d’ Histoire Naturelle, Paris, France; NMPC: National Museum (Natural History), Prague, Czech Republic.

## Taxonomy

### Family Buthidae C.L. Koch, 1837

#### Genus Lychas C.L. Koch, 1845

##### Lychas mucronatus

(Fabricius, 1798)

Figures 1 9

Scorpio mucronatus  Fabricius 1798: 294.
Scorpio armillatus  Gervais 1841: 284 (synonymized by Thorell 1888: 330).
Scorpio (Androctonus) curvidigitatus  Gervais 1843: 129 (synonymized by Thorell 1893: 368).
Tityus varius  C.L. Koch 1844 (synonymized by Thorell 1888: 330).
Isometrus chinensis  Karsh 1879: 116 (synonymized by Kraepelin 1891: 81).
Isometrus atomarius  Simon 1884: 363 (synonymized by Kraepelin 1891: 81).
Lychas baldasseronii  Caporiaco 1947: 247 (synonymized by Kovařík 1997: 342).
Lychas mentaweius  Roewer 1943: 212 (synonymized by Kovařík 1997: 342).
Lychas nucifer  Basu 1964: 100 (synonymized by Kovařík 1997: 342).
Lychas mucronatus Pocock 1900: 36-37; Kovařík 1997: 341–344, Figs 10, 12, 29, 31, 80-82, 93, 98; Fet and Lowe 2000: 164, 165 [detailed reference list until 1998].

###### Type locality.

India orientali, UZMD.

###### Type material.

Lost.

###### Material examined.

Shidian District, 17/VIII/2010, Dahe Jiang, Chaowu Yang and Zhiyong Di leg, 11 females, 3 males, 2 juveniles (MWHU, Ar.-MWHU-YNSD1010–15); Shidian District (24.42°N, 99.24°E), VIII/2008, Heng Xiao leg, 7 females, 7 males, 6 immatures (MWHU, Ar.-MWHU-YNSD0801–20); Longling District (24.47°N, 98.56°E), 18/VIII/2010, Wenxin Li, Hui Liu, Xiaohua He and Zizhong Yang leg, 14 females, 3 males, 4 juveniles (MWHU, Ar.-MWHU-YNLL1010–20); Gengma District, 6/VIII/2004, Zizhong Yang and Yuhua Yang leg, 2 males (BHDC, Ar.-BHDC-YNGM0401–02); Yun District, 21/VII/2003, Zizhong Yang and Benyong Mao leg, 2 males (BHDC, Ar.-BHDC-YNYX0301–02); Yongde District, 20/VII/2009, Benyong Mao leg, 1 female, 3 males (BHDC, Ar.-BHDC-YNYD0901–04); Mojiang District, Tongguan town, 22/XI/2010, Dongming Luo leg, 2 males, 5 females, 1 juvenile (MWHU, Ar.-MWHU -YNMJ1001–08).

###### Diagnosis.

**Figures 1–4.**
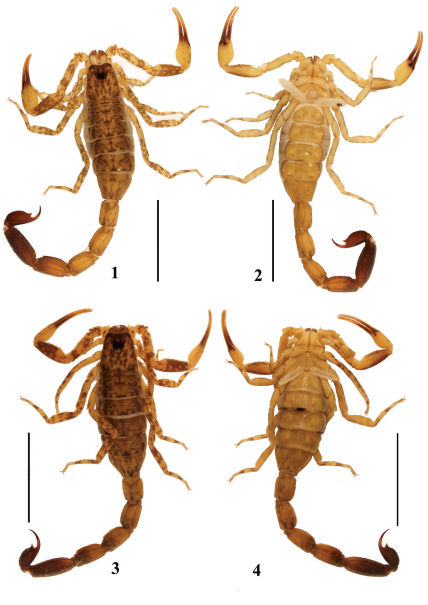
Habitus of Lychas mucronatus. **1–2** Male (Ar.-MWHU-YNSD1010), dorsal and ventral views **3–4** Female (Ar.-MWHU-YNSD1011), dorsal and ventral views. Scale bars: 12.0 mm.

**Figures 5–9.**
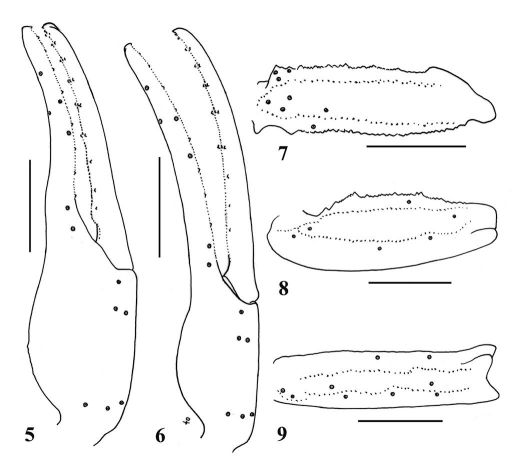
5, 7–9.Lychas mucronatus. Male (Ar.-MWHU-YNSD1010): **5** Chela, dorsal aspect **7** Femur dorsal aspect **8–9** Patella dorsal and external aspects. Scale bars: 2.0 mm. **6** Lychas mucronatus, female (Ar.-MWHU-YNSD1011). Chela, dorsal aspect. Scale bars: 2.0 mm.

(Modified from Kovařík 1997). Total length about 40–65 mm in males and females (Figs 1–4). Male differs from female in having fingers of pedipalps proximally twisted (Fig. 5). Sixth cutting edge on movable and fixed fingers of pedipalps, usually with 3 external granules each (rarely 2 or 4 granules). First and second metasomal segments with 10 carinae, third and fourth segments with eight carinae. Ventral surface of seventh mesosomal segment with two carinae (not always discernible). Position and distribution of trichobothria on pedipalps as figures 5–9.

From its general morphology, Lychas mucronatus certainly related to Lychas krali Kovařík, 1995, described from Umphang River in Thailand. They have same important characters: second segment of metasoma with ten carinae, third metasomal segment with eight carinae; sixth cutting edge on movable fingers of pedipalps with two to four external granules; legs spotted. Lychas mucronatus can be distinguished from Lychas krali by the following characters: manus of pedipalps bright yellow with sparse, minute black spots, patella predominantly dark, compared with Lychas krali, in which the manus of pedipalps have the same color as patella and femur; pectinal teeth number 16–26, pectinal teeth 10–19 in Lychas krali; metasoma of approximately the same length in both sexes in Lychas mucronatus, whereas the metasoma much longer in males than in females in Lychas krali (Kovařík 1997: 360).

###### Ecology.

This species is common. We collected from mixed forest and buzzed canebrake. They are found in the bark, the gap of soil and under the stones.

###### Distribution.

Cambodia, China (Guangxi, Hainan and Yunnan), India, Indonesia, Laos, Malaysia, Myanmar, Philippines, Thailand, and Vietnam (Kovařík 1997; Zhu et al. 2004).

### Family Euscorpiidae Laurie

#### Subfamily Scorpiopinae Kraepelin, 1905

##### Euscorpiops Vachon, 1980

###### Euscorpiops kubani

Kovařík, 2004 rec. n.

**Figures 10–13.**
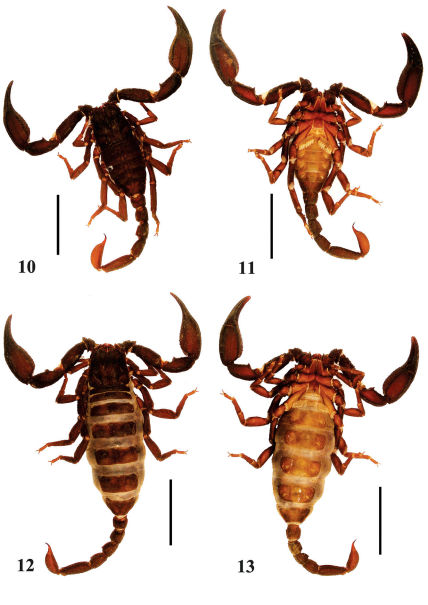
Habitus of Euscorpiops kubani. **10–11** Male (Ar.-MWHU-YNMH1001), dorsal and ventral views **12–13** Female (Ar.-MWHU-YNMH1002), dorsal and ventral views. Scale bars: 12.0 mm.

Figures 10 [Fig F4] 28

Euscorpiops kubani Kovařík 2004: 13–18, Figs 1–6, tab. 1.
Euscorpiops kubani : Kovařík 2005: 1–10, Figs 1–6, tab. 1.

####### Type locality.

Laos, prov. Phongsaly, Phongsaly env.

####### Type material.

Holotype male, Laos, prov. Phongsaly, Phongsaly env., 21°41'2"N, 102°06'8"E, 1500 m, Vt KubÆ leg, (deposited in the Moravian Museum, Brno, Czech Republic). Other type materials. Allotype female: Laos, prov. Phongsaly, Phongsaly env., 21°41'2"N, 102°06'8"E, 1500 m, 28/V/20/VI/2003, leg. Vt KubÆ; (Moravian Museum, Brno, Czech Republic). 1 paratype male, Laos, prov. Phongsaly, Ban Sano Mai env., 21°21'N, 102°03'E, ca 1150 m, 19. 26/V/2004, Vt KubÆ leg, (FKCP, followed Kovařík 2004).

####### Material examined.

Menghai District (21.99°N, 100.45°E), 21/VIII/2010, Wenxin Li, Xiaohua He, Hui Liu, Dahe Jiang and Zhiyong Di leg, 3 females, 3 males, 3 female immatures, 2 male immatures (MWHU, Ar.-MWHU- YNMH 1001– 11); Menghai District, 21/VIII/2006, Bin Xu leg, 1 female immature (BHDC, Ar.-BHDC- YNMH 0601–02).

####### Diagnosis.

(Modified from Kovařík 2004) Adult 39–50 mm. Mainly color uniformly reddish-black. Pectinal teeth number 6–8. Sexual dimorphism expressed in shape of pedipalp fingers: in male flexed, in female nearly straight (slightly undulate). Pedipalp patella with 18 or rarely 19 external trichobothria (5 or 6 *eb*, 2 *esb*, 2 *em*, 4 *est*, 5 *et*) (Kovařík 2004), and 9 or 10 ventral trichobothria.

Euscorpiops kubani is similar to Euscorpiops shidian Qi, Zhu and Lourenço, 2005 in shape and color (Figs 10–13, 50–53): both are characterized by the presence of 18 trichobothria (Euscorpiops kubani: mainly with 18) on the external surface of pedipalp patella, 6–8 pectinal teeth, chela with similar length/width ratio (Tab. 2). Both species can be separated by: male pedipalp chela fingers strongly scalloped in Euscorpiops kubani, whereas in Euscorpiops shidian males are slightly scalloped or straight, being the lobe and corresponding notch are reduced to absent; pectinal fulcra small, reduced or absent in Euscorpiops kubani, but well developed in Euscorpiops shidian.

####### Description

(based on specimens: Ar.-MWHU-YNMH1001–02).

######## Coloration:

Carapace dark red brown. Median and lateral ocular tubercles black. Tergites mostly dark red brown to dark brown. Metasoma segments dark red brown to dark brown. Vesicle red brown with a reddish aculeus. Chelicerae yellow brown with fingers dark red brown gradually lighter toward the tip. Pedipalp femur and patella dark red brown, chela manus and fingers red brown. Legs red brown with yellow brown tarsi. Tarsal ungues yellowish brown. Sternum, genital operculum and sternites pale brown. Pectines yellowish.

######## Morphology.

*Prosoma*: Carapace with sparse, coarse granules; lateral furrow broad; anterior median furrow broad and moderately deep; posterior median furrow deep; margin behind lateral eyes with granules, other margins smooth. Median eyes situated anteriorly compared to center of carapace; three pairs of lateral ocelli, posterior smallest. Median ocular tubercle with granules and a pair of big median eyes and a median furrow. Lateral ocular tubercle with some granules around eyes.

######## Mesosoma:

Tergites sparsely covered with coarse granules, posterior part of tergites with bigger granules; tergites II–VI with a median carina; tergite VII with two pairs of lateral carinae (with bigger granules). Pectinal teeth count 6–8, fulcra small reduced to absent. Genital operculum subtriangular. Sternites smooth and shiny; segment VII with 4 weak ventral carinae and few granules.

######## Metasoma:

Tegument coarse. Segments II to V longer than wide; segments I to V with respectively 10-8-8-8-7 carinae, segments II–IV with a pair of vestigial lateral carinae; all dorsal carinae crenulate, slightly stronger distally; segment V carinae with smaller granules dorsally and larger serration ventrally. Vesicle with few setae and granules.

######## Pedipalps:

**Figures 14–21. F4:**
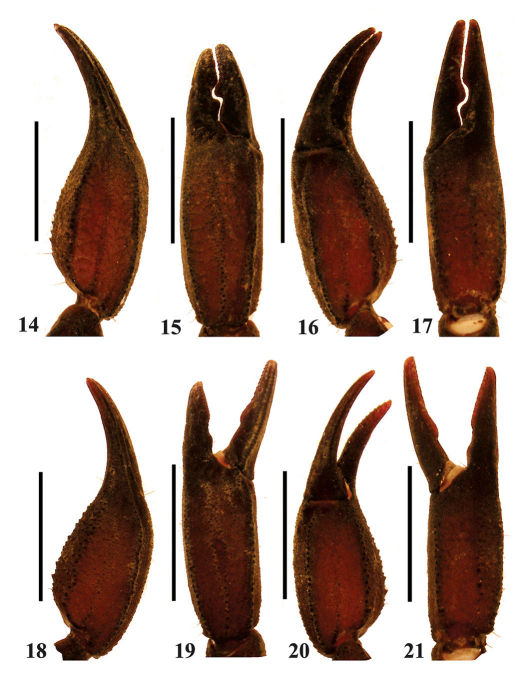
Euscorpiops kubani. **14–17** Male (Ar.-MWHU-YNMH1001). Chela dorsal, external, ventral and internal aspects **18–21** Female (Ar.-MWHU-YNMH1002). Chela dorsal, external, ventral and internal aspects.Scale bars: 6.0 mm.

Tegument coarse. Femur with external, dorsointernal, dorsoexternal, ventrointernal, ventroexternal and internal carinae granulated; tegument with scattered granules dorsally and smooth ventrally. Patella with dorsointernal, dorsoexternal, ventrointernal, ventroexternal and external carinae with big granules; two large spinoid granules present on the internal aspect; tegument with smooth granules dorsally and ventrally. Trichobothrial pattern C, neobothriotaxic (Vachon 1974); patella with 18 (rarely 19) external trichobothria (5 or 6 *eb*, 2 *esb*, 2 *em*, 4 *est*, 5 *et*) (Kovařík 2004), 10 or 9 ventral trichobothria (Fig. 27). Chela with length/width ratio: 2.7–3.0 in adult males and 2.7–2.9 in females (3.1 on male holotype, 3.2 on female paratype after Kovařík 2004). Chela with dorsal marginal, external secondary, and ventrointernal carinae granulated (Figs 14–21); ventrointernal carina with some big granules; tegument with granules forming reticulated pattern; male fingers scalloped with a pronounced lobe in the movable finger and a corresponding notch in fixed finger, lobe and corresponding notch reduced to absent in females (Figs 23, 25).

######## Chelicerae:

Tegument smooth. Tibia smooth. Movable finger with 4 teeth on dorsal edge, 6–7 teeth (not constant) on ventral edge. Fixed finger with 3 teeth on dorsal edge.

######## Legs:

Tegument coarsely granular dorsally, except basitarsi and telotarsi, smooth ventrally. Trochanters with few setae. Femur dorsal surface with few small granules, external surface with a granular carina, internal surface with two granular carinae. Patella internally with a dentate carina. Tibia with few setae and small granules, without spurs. Basitarsi with some spinules, few setae and 2 lateral pedal spurs. Tarsi ventrally with one row of short spinules and few setae. Tarsal ungues curved and hook-like.

**Figures 22–28.**
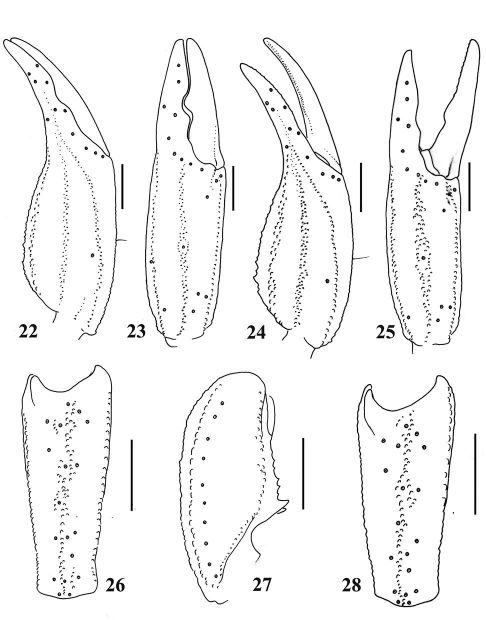
Euscorpiops kubani. **22–23** Male (Ar.-MWHU-YNMH1001): Chela dorsal and external aspects **26–27** Male (Ar.-MWHU-YNMH1001): Patella external and ventral aspects **24–25** Female (Ar.-MWHU-YNMH1002): Chela dorsal and external aspects **28** Female (Ar.-MWHU-YNMH1002): Patella external aspect. Scale bars: 2.0 mm.

####### Variation.

Female and male paratypes: coloration and morphology are very similar to holotype (Kovařík 2004). Sexual dimorphism: adult males, with more pronounced lobes on the movable fingers of the chela, and a more pronounced notch in the fixed finger and bigger pectinal teeth than females. Measurements in table 1. Feature datasets in table 2.

**Table 1.** Measurements (in mm) of Euscorpiops kubani (Ar.-MWHU-YNMH1001 and Ar.-MWHU – YNMH 1002), Euscorpiops shidian (Ar.-BHDC-YNSD0401 and Ar.-MWHU-YNSD1001) and Euscorpiops xui (Ar.-BHDC- YNML 0901 and Ar.-BHDC- YNML0902).

	Euscorpiops kubani		Euscorpiops shidian		Euscorpiops xui	
	Male	Female	Male	Female	Male	Female
Total length:	47.0	48.0	52.4	54.8	56.3	57.5
Carapace:						
-Length	7.6	7.1	7.9	8.3	8.4	9.2
-Anterior width	4.5	4.5	4.9	4.7	5.0	5.3
-Posterior width	7.7	7.5	9.0	8.7	8.9	8.3
Mesosomal segments: -Length	11.5	15.5	17.1	18.0	18.1	19.2
Metasomal segment I: -Length	2.5	2.9	3.0	3.0	3.1	3.0
-Width	2.9	2.8	3.0	3.1	3.3	3.4
-Depth	2.4	2.2	2.3	2.6	2.6	2.7
Metasomal segment II: -Length	3.0	3.0	3.1	3.3	3.5	3.3
-Width	2.6	2.5	2.7	2.6	3.0	3.1
-Depth	2.2	2.0	2.3	2.3	2.7	2.5
Metasomal segment III : -Length	3.5	3.2	3.5	3.7	3.8	3.7
-Width	2.5	2.3	2.5	2.5	2.7	2.9
-Depth	2.2	2.2	2.4	2.3	2.5	2.5
Metasomal segment IV: -Length	4.3	3.4	3.8	4.3	4.3	4.3
-Width	2.4	2.2	2.4	2.2	2.5	2.6
-Depth	2.2	2.3	2.4	2.3	2.4	2.5
Metasomal segment V: -Length	6.9	5.9	6.5	7.0	6.7	7.0
-Width	2.3	2.2	2.2	2.2	2.4	2.5
-Depth	2.2	2.0	2.3	2.2	2.4	2.4
Telson: -Length	7.8	6.8	7.6	7.4	8.4	7.9
-Width	2.5	2.2	2.4	2.2	2.6	2.2
-Depth	2.2	1.8	2.2	2.0	2.5	2.2
Pedipalp femur: -Length	7.9	6.4	8.9	8.6	10.4	8.7
-Width	3.0	2.6	3.3	3.1	3.2	3.5
-Depth	2.4	2.3	2.5	2.6	2.7	2.8
Pedipalp patella: -Length	7.1	6.2	7.6	7.4	9.1	7.9
-Width	3.6	3.3	4.1	4.0	4.4	4.8
-Depth	2.9	2.7	2.9	2.9	3.2	3.3
Chela: -Length	14.5	13.5	16.0	16.5	19.5	17.0
-Width (manus)	4.6	4.4	4.7	4.7	4.7	5.3
-Depth (manus)	3.8	3.4	3.6	3.6	4.0	4.1
Movable finger: -Length	7.7	7.0	8.2	8.9	8.8	8.8
Pectinal teeth (L/R)	8/8	7/6	8/8	8/8	8/8	7/7

**Table 2.** Feature datasets of specimens of Euscorpiops kubani, Euscorpiops shidian and Euscorpiops xui**.** BL, body length; VTPP, ventral trichobothria of pedipalp patella (L/R); ETPP, external trichobothria of pedipalp patella (L/R); LWRC, length/width ratio of chela; PT, pectinal teeth; im, immature; HT, holotype; PT, paratype.

Species	Serial number	Sex	BL≈	VTPP	ETPP	PT	LWRC
E. kubani	HT(FKCP)	♂	39	10/10	19/19	8/8	3.1
	PT(FKCP)	♀	44	10/10	18/18	7/7	3.2
	Ar.-MWHU-YNMH1001	♂	47	10/10	18/18	8/8	3.2
	Ar.-MWHU-YNMH1002	♀	48	9/9	18/18	7/6	3.1
	Ar.-MWHU-YNMH1003	♂	48	10/10	18/18	8/8	3.1
	Ar.-MWHU-YNMH1004	♂	44	9/10	18/16†	6/7	2.9
	Ar.-MWHU-YNMH1005	♂im		10/10	18/18	7/7	
	Ar.-MWHU-YNMH1006	♂im		10/9	18/18	8/8	
	Ar.-MWHU-YNMH1007	♀	45	10/10	18/18	6/6	2.9
	Ar.-MWHU-YNMH1008	♀	44	9/10	18/18	7/6	2.7
	Ar.-MWHU-YNMH1009	♀im		9/10	18/18	7/6	
	Ar.-MWHU-YNMH1010	♀im		9/9	18/18	7/7	
	Ar.-MWHU-YNMH1011	♀im		10/10	18/18	6/6	
	Ar.-BHDC-YNMH0601	♀	45	9/10	17/17	6/6	2.7
	Ar.-BHDC-YNMH0601	♀im		11/11	18/18	6/6	
E. shidian	HT(MHBU)	♂	49	11/11	17/17§	7/7	1.6#
	PT(MHBU)	♂	60	11/11	17/17§	7/7	2.4#
	Ar.-BHDC-YNSD0401	♂	52	11/11	18/18	8/8	3.4
	Ar.-MWHU-YNSD1001	♀	55	10/11	18/18	8/8	3.5
	Ar.-MWHU-YNSD1002	♂	47	11/11	18/18	8/7	3.5
	Ar.-MWHU-YNSD1003	♂	50	11/11	18/18	8/8	3.3
	Ar.-MWHU-YNSD1004	♂im		11/11	18/18	8/8	
	Ar.-MWHU-YNSD1005	♀	50	12/12	18/18	6/6	3.2
	Ar.-MWHU-YNSD1006	♀	45	11/11	18/18	8/7	3.2
	Ar.-MWHU-YNSD1007	♀	45	11/11	18/18	6/7	3.5
	Ar.-MWHU-YNSD1008	♀	50	11/11	18/18	8/7	3.2
E. xui	HT(MHBU)	♀	66	10/10	19/19	7/7	3.6
	PT(MHBU)	♂	54	10/10	19/19	8/8	4.1
	Ar.-BHDC-YNML0901	♂	56	10/10	18/19	8/8	4.0
	Ar.-BHDC-YNML0902	♀	58	10/10	19/19	7/7	3.4

**†** It is visible that the right patella of pedipalp of this specimen (Ar.-MWHU-YNPMH1004) didn’t developed well, in respect that with external trichobothria *et4* and *est2* absent (the position and terminology followed Kovařík 2000:157). **§** As these specimens came from the same village, it is very puzzling that with obvious external trichobothria difference. **#** Maybe there are different methods of measurement adopted by these authors lead to an enormous difference among length/width ratio of chela of type specimens and new material, however, it is obvious that they are with the same shape (Figs 50–61; Qi et al. 2005, Figs 78–83).

**Figures 29–32.**
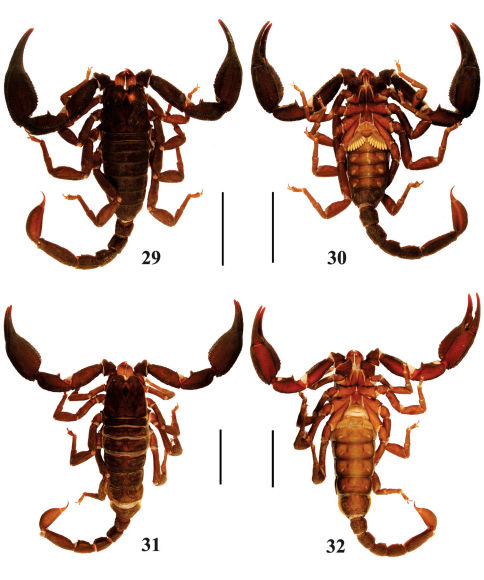
Habitus of Euscorpiops puerensis. **29–30** Male paratype (Ar.-MWHU-YNPE0805), dorsal and ventral views **31–32** Female holotype (Ar.-MWHU-YNPE0801), dorsal and ventral views. Scale bars: 12.0 mm.

**Figures 33–40. F7:**
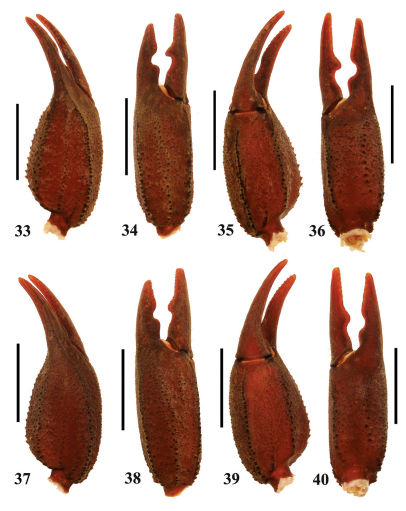
Euscorpiops puerensis. **33–36** Male paratype (Ar.-MWHU-YNPE0805). Chela dorsal, external, ventral and internal aspects **37–40** Female holotype (Ar.-MWHU-YNPE0801). Chela dorsal, external, ventral and internal aspects.Scale bars: 6.0 mm.

**Figures 41–49.**
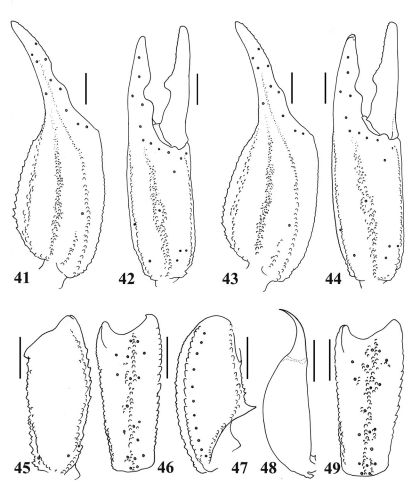
41–42, 45–48Euscorpiops puerensis. Male paratype (Ar.-MWHU-YNPE0805): **41–42** Chela dorsal and external aspects **45** Femur dorsal aspect **46–47** Patella external and ventral aspects **48** Telson, lateral aspect. Scale bars: 2.0 mm. **43–44, 49.** Euscorpiops puerensis. Female holotype (Ar.-MWHU-YNPE0801): **43–44** Chela dorsal and external aspects **49** Patella external aspect. Scale bars: 2.0 mm.

####### Ecology.

This species was collected from moist mixed forest and village. They are found in the shambles (brick or stones) and under the clod.

####### Distribution.

China (Yunnan), Laos.

###### Euscorpiops puerensis

Di, Wu, Cao, Xiao & Li, 2010

Figures 29 [Fig F7] 49

Euscorpiops puerensis Di et al. 2010b: 49–61, Figs 1–34, tabs. 1–2.

####### Type locality.

China,Yunnan, Puer.

####### Type materials, examined.

Female holotype, China: Yunnan, Puer, X/2008, Heng Xiao leg, (Ar.-MWHU-YNPE0801); paratypes: 5 males and 4 females (including 2 male immatures and 1 female immature) (Ar.-MWHU-YNPE0802–06, Ar.-MWHU-YNPE0807–10), same data as holotype.

####### Diagnosis.

Euscorpiops puerensis differs from all other species in the genus on the basis of the following combination of characters: 18 external trichobothria (5 *eb*, 2 *esb*, 2 *em*, 4 *est*, 5 *et*), and 10 or 11 ventral trichobothria in the pedipalp patella (10 specimens); chela with a length/width ratio average of 2.7 in males and females (5 males and 2 females); pedipalp chela fingers of adult females and males scalloped; pectinal teeth count 7–8; pectinal fulcra present.

Euscorpiops puerensis appears to be closely related to Euscorpiops vachoni Qi, Zhu and Lourenço, 2005: both are medium-sized scorpions characterized by the presence of 10 or 11 trichobothria on the ventral surface of pedipalp patella, a pronounced lobe on the movable finger and a corresponding notch on fixed finger of adult males, 7–8 pectinal teeth. The shape of the chela manus provides the most pronounced difference between them, in Euscorpiops puerensis is flat dorsoventrally, whereas it is short, stout, and robust in Euscorpiops vachoni. Euscorpiops puerensis may be distinguished from Euscorpiops kubani and Euscorpiops sejnai Kovařík 2000 by means of the following features: pedipalp chela fingers are distinctly scalloped on adult males and females in Euscorpiops puerensis, whereas in Euscorpiops kubani chela fingers are scalloped on male and nearly straight on female, and in Euscorpiops sejnai male chela fingers are slightly scalloped (female unknown); 10–11 trichobothria on ventral surface of patella in Euscorpiops puerensis, whereas there are 9 in Euscorpiops sejnai, and 9–10 in Euscorpiops kubani (11 rarely); chela with a length/width ratio average of 2.7 on males and females, whereas in Euscorpiops kubani is higher than 2.7, and on Euscorpiops sejnai is 2.75; 7–8 pectinal teeth, whereas there are 4–7 in Euscorpiops sejnai, 6–8 in Euscorpiops kubani; total length 48.8 to 60.0 mm in Euscorpiops puerensis, whereas both Euscorpiops sejnai and Euscorpiops kubani are smaller than 48.0 mm (Kovařík 2000, 2004, 2005).

####### Description.

See Di et al. (2010b).

####### Ecology.

This species is found under the stones in mixed forest.

####### Distribution.

China (Yunnan, just the type locality).

###### Euscorpiops shidian

Qi, Zhu & Lourenço, 2005

**Figures 50–53.**
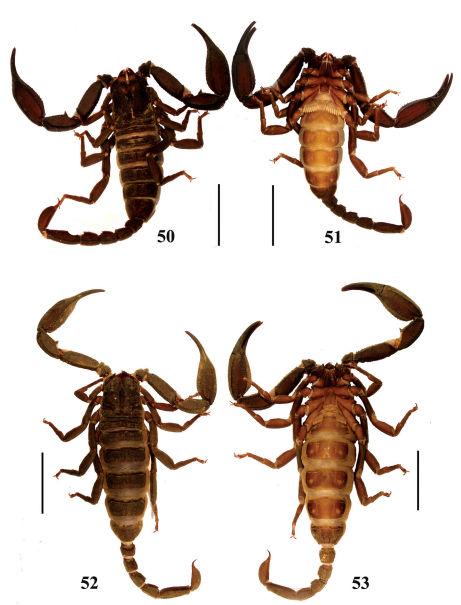
Habitus of Euscorpiops shidian. **50–51** Male (Ar.-BHDC-YNSD0401), dorsal and ventral views **52–53** Female (Ar.-MWHU-YNSD1001), dorsal and ventral views. Scale bars: 12.0 mm.

Figures 50 [Fig F10] 68

Euscorpiops shidian Qi et al. 2005: 18, 22–25, Figs 78–93.

####### Type locality.

China, Yunnan Province, Shidian District.

####### Type material.

Holotype, male, Yunnan Province, Shidian District, Jiucheng town (24.43°N, 99.09°E), 15/VI/2004, Yingda Zhang and Zizhong Yang leg, (MHBU); paratypes: 1 female (MNHN), 2 females (MHBU), same data as holotype.

####### Material examined.

Shidian District, Jiucheng town (24.43°N, 99.09°E), 16/VIII/2010, Dahe Jiang and Zhiyong Di leg, 5 females, 2 males, 1 male immature, 1 juvenile (MWHU, Ar.-MWHU-YNSD1001–09); Shidian District, Jiucheng town (24.43°N, 99.09°E), 15/VI/2004, Yingda Zhang and Zizhong Yang leg, 1 male, 1 juvenile (BHDC, Ar.-BHDC-YNSD0401–02), same data as holotype.

####### Diagnosis.

Euscorpiops shidian differs from all other species in the genus on the basis of the following combination of characters: pedipalp patella with 18 external (5 *eb*, 2 *esb*, 2 *em*, 4 *est*, 5 *et*), and 10–12 ventral trichobothria (rarely 10 or 12); chela with length/width ratio average of 3.3 (5 males and 5 females); inner surface of pedipalp chela fingers on adult females and males nearly straight; pectinal fulcra present (few and small).

Euscorpiops shidian is morphologically most similar to Euscorpiops kubani. Both species are characterized by the presence of 18 trichobothria (Euscorpiops kubani: mainly with 18) on the external surface of pedipalp patella, 6–8 pectinal teeth, chela with similar length/width ratio (Tab. 2). They can be separated by: male pedipalp chela fingers slightly scalloped or straight in Euscorpiops shidian, whereas in Euscorpiops kubani males they are strongly scalloped; pectinal fulcra few but obvious in Euscorpiops shidian, pectinal fulcra small reduced to absent in Euscorpiops kubani.

Euscorpiops shidian may be separated from Euscorpiops puerensis, Euscorpiops vachoni and Euscorpiops validus Di, Cao, Wu and Li, 2010 on the basis of the following character: chela slender with a length/width ratio average of 3.3, whereas in Euscorpiops puerensis chela with a length/width ratio average of 2.7, and in Euscorpiops vachoniand Euscorpiops validus chela smaller than 3.0; Euscorpiops shidian may be separated from Euscorpiops yangi Zhu, Zhang and Lourenço, 2007 and Euscorpiops xui Sun and Zhu, 2010 by the following character: patella of pedipalp with 11 ventral trichobothria (rarely 10 and 12, Table 2), whereas on Euscorpiops yangi with9–10 (Zhu et al. 2007), on Euscorpiops xui with 10 (4 specimens, Table 2); patella of pedipalp with 18 external trichobothria whereas on Euscorpiops xui with 18–19.

####### Description

(based on male (Ar.-BHDC-YNSD0401) and female (Ar.-MWHU-YNSD1001))**.**

######## Coloration:

Carapace dark red black brown. Median and lateral ocular tubercles black. Tergites mostly dark red brown to dark brown. Metasoma segments dark red brown to dark brown; telson, vesicle brown, aculeus redish. Chelicerae yellow brown, fingers red brown gradually lighter toward the tip. Pedipalp femur and patella dark brown, chela manus and fingers dark red brown. Legs red brown with yellow brown tarsi. Tarsal ungues yellowish brown. Sternum, genital operculum and sternites brown. Pectines yellowish.

######## Morphology.

*Prosoma*: Tegument coarse with fine and smooth granules. Carapace with sparse, fine granules; lateral furrow broad; anterior median furrow broad and moderately deep; posterior median furrow deep; margin behind lateral eyes with granules, other margins smooth. Median eyes situated anteriorly respect to the center of carapace; three pairs of lateral ocelli, posterior smallest. Median ocular tubercle coarse with granules and a pair of big median eyes and a median furrow. Lateral ocular tubercle with some granules around eyes.

######## Mesosoma:

Tergites densely covered with fine granules, posterior part of tergites with bigger granules; tergite II to tergite VI with a median carina; tergite VII with two pairs of lateral carinae. Pectinal teeth count 6–8, fulcra small and obvious. Genital operculum subtriangular. Sternites smooth; segment VII with four weak ventral carinae with granules.

######## Metasoma:

Tegument coarse. Segments II to V longer than wide; segments I to V with respectively 10-8-8-8-7 carinae, segments II–IV with a pair of vestigial lateral carinae; dorsal carinae crenulated, slightly stronger distally; on segment V carinae with smaller granules dorsally and larger serration ventrally. Vesicle with sparse small granules, and few setae.

######## Pedipalps:

**Figures 54–61. F10:**
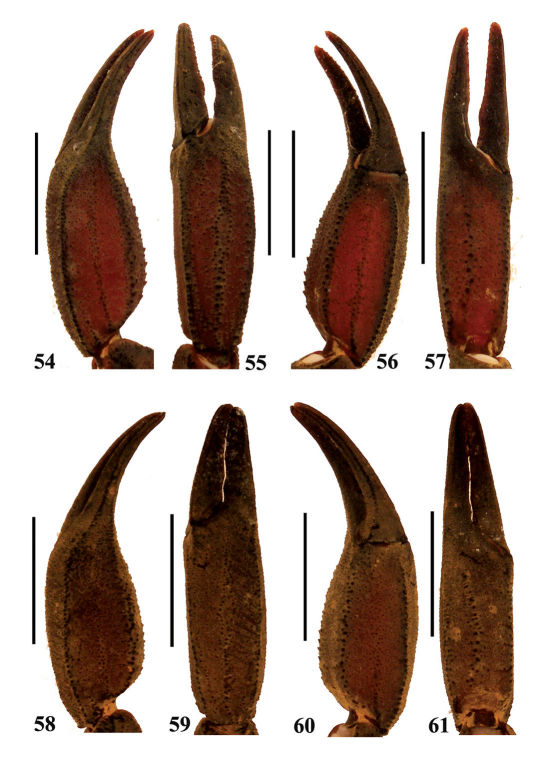
Euscorpiops shidian **54–57** Male (Ar.-BHDC-YNSD0401). Chela (left) dorsal, external, ventral and internal aspects **58–61** Female (Ar.-MWHU-YNSD1001). Chela (left) dorsal, external, ventral and internal aspects.Scale bars: 6.0 mm.

Tegument coarse with fine and smooth granules. Femur with external, dorsointernal, dorsoexternal, ventrointernal, ventroexternal and internal carinae granulated; tegument with scattered granules dorsally and smooth ventrally. Patella with dorsointernal, dorsoexternal, ventrointernal, ventroexternal and external carinae with big granules; two large spinoid granules present on the internal aspect; tegument with smooth granules dorsally and ventrally. Trichobothrial pattern C, neobothriotaxic (Vachon1974); patella with 18 external trichobothria (5*eb*, 2 *esb*, 2 *em*, 4 *est*, 5 *et*), 11 (rarely 10 and 12) ventral trichobothria (Fig. 67). Chela with a length/width ratio average of 3.3 on adult males and females. Chela with dorsal marginal, external secondary, and ventrointernal carinae granulated (Figs 54–61); ventrointernal carina with some big granules; tegument with small granules forming reticulated pattern; fingers nearly straight (Figs 63, 65).

######## Chelicerae:

Tegument smooth. Tibiae smooth. Movable finger with 4 teeth on dorsal edge and 6–7 teeth (not constant) on ventral edge. Fixed finger with 3 teeth on dorsal edge.

######## Legs:

Tegument coarse dorsally except basitarsi and telotarsi, smooth ventrally. Trochanters with few setae. Femur dorsal surface with some small granules, external surface with one granular carina, internal surface with two granular carinae. Patella internally with one dentate carina. Tibia with few setae and small granules, without spurs. Basitarsi with some spinules, few setae and two lateral pedal spurs. Tarsi ventrally with one row of short spinules and few setae. Tarsal ungues curved and hook-like.

**Figures 62–68.**
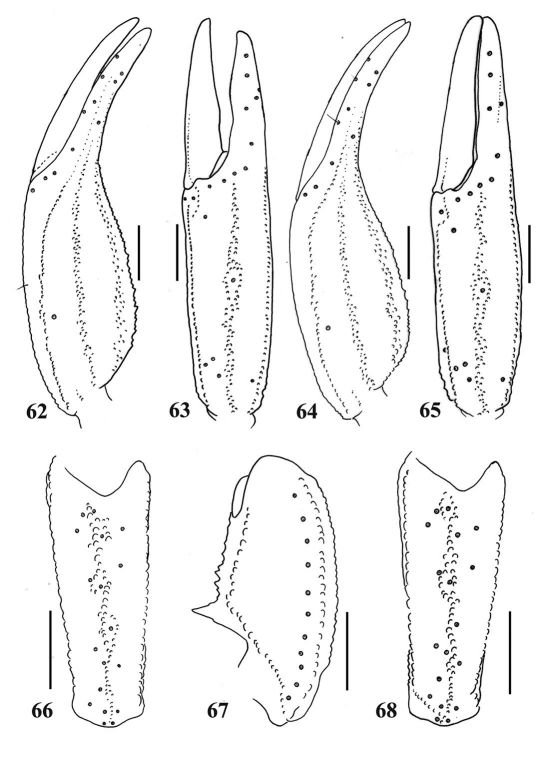
62–63, 66–67.Euscorpiops shidian. Male (Ar.-BHDC-YNSD0401): **62–63** Chela (left) dorsal and external aspects **66–67** Patella (left) external and ventral aspects. Scale bars: 2.0 mm **64–65, 68.** Euscorpiops shidian. Female (Ar.-MWHU-YNSD1001) **64–65** Chela (left) dorsal and external aspects **68** Patella (left) external aspect. Scale bars: 2.0 mm.

####### Variation.

Female and male paratypes: coloration and morphology are very similar to holotype (see Qi et al. 2005). Sexual dimorphism: the pectinal teeth of adult males are clearly bigger than those of adult females; this sexual dimorphism is common in Euscorpiops and Scorpiops. Measurements in table 1. Feature datasets in table 2.

####### Ecology.

This species was collected from moist mixed forest and hamlets. They are found on the wall in the night and under stones in the day.

####### Distribution.

China (Yunnan).

###### Euscorpiops vachoni

Qi, Zhu & Lourenço, 2005

Figures 69–72

Euscorpiops vachoni  Qi et al. 2005: 18–21, Figs 62–77.

####### Type locality.

China, Yunnan Province, Mengla District.

####### Type material.

Holotype male. Mengla district (21°29'N, 101°33'E), Yunnan Province, 2/VIII/2004, Zizhong Yang, Jing Li and Caixia Yuan leg, (MHBU); Paratypes: 1 female, same data as holotype (MHBU), 1 male, Tibet, Nyingchi district, 3/VIII/2003, Feng Zhang leg, (MNHN).

####### Material examined.

Mengla District, 2/VIII/2004, Zizhong Yang, Jing Li and Caixia Yuan leg, 1 male immature, same data as holotype (BHDC, Ar.-BHDC-YNML0401).

####### Diagnosis.

(Modified from Qi et al. 2005) Euscorpiops vachoni differs from all other species of the genus on the basis of the following combination of characters: yellow brown color, 18 (17 in Qi et al. 2005) external trichobothria (5 *eb*, 2 *esb*, 2 *em*, 4 *est*, 5 *et*), and 10 ventral trichobothria in the pedipalp patella; adult chela manus stout and rounded (see Qi et al. 2005, Figs 63–66); pedipalp chela fingers on adult males scalloped; pectinal teeth: 7–8.

Euscorpiops vachoni appears to be closely related to Euscorpiops puerensis: both are medium-sized scorpions, characterized by the presence of 10 or 11 trichobothria on the ventral surface of pedipalp patella, a pronounced lobe on the movable finger and a corresponding notch on fixed finger of adult males, 7–8 pectinal teeth. The most pronounced difference between both species is: chela manus short, stout, and robust in Euscorpiops vachoni, whereas it is flat dorsoventrally in Euscorpiops puerensis.

Euscorpiops vachoni may be separated from Euscorpiops shidian and Euscorpiops yangi on the basis of the following character: chela with a length/width ratio smaller than 3.0, whereas in Euscorpiops shidian higher than 3.2, and in Euscorpiops yangi 3.4 (males) and 3.3 (females). Euscorpiops vachoni may be separated from Euscorpiops kubaniand Euscorpiops validus by the following characters:yellow brown color in Euscorpiops vachoni, compared with dark red brown in Euscorpiops kubani, and dark brown inn Euscorpiops validus; chela manus stout and rounded, whereas in Euscorpiops kubani and Euscorpiops validus flat*. E. vachoni* may be separated from Euscorpiops xui by the following characters: patella of pedipalp with 18 external trichobothria whereas in Euscorpiops xui with 18–19; chela with a length/width ratio smaller than 3.0, whereas in Euscorpiops xui with a length/width ratio higher than 3.4.

####### Description.

See Qi et al. (2005).

**Figures 69–72.**
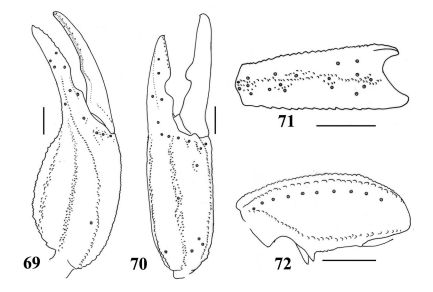
Euscorpiops vachoni. **69–70, 71** Holotype, male (MHBU, followed Qi et al. 2005) **69–70** Chela dorsal and external aspects **71** Patella ventral aspect. Scale bars: 2.0 mm. **72**Male (immature, Ar.-BHDC-YNML1001). Patella external aspect. Scale bars: 2.0 mm.

####### Ecology.

This species is uncommon, type materials collected from moist mixed forest close to the border of China and Laos.

####### Distribution.

China (Yunnan, just the type locality).

####### Notes.

The immature male specimen checked bears 18/18 external trichobothria (5 *eb*, 2 *esb*, 2 *em*, 4 *est*, 5 *et*), and 10/10 ventral trichobothria in the pedipalp patella, 17 external trichobothria and 10 ventral trichobothria in the pedipalp patella on holotype (see Qi et al. 2005: 18).

###### Euscorpiops validus

Di, Cao, Wu & Li, 2010

**Figures 73–76.**
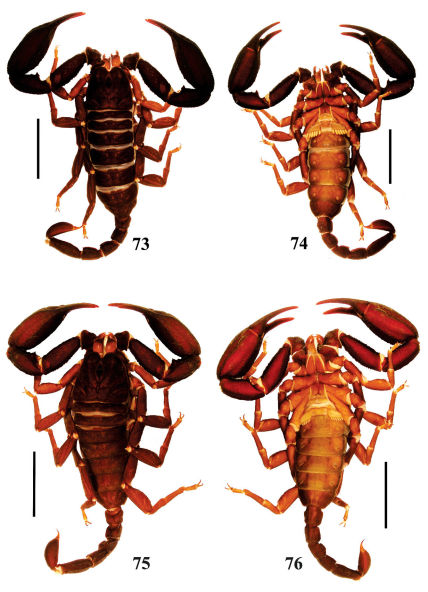
Habitus of Euscorpiops validus. **73–74** Male holotype (Ar.-MWHU-YNHH0901), dorsal and ventral views **75–76** Female allotype (Ar.-MWHU-YNHH0902), dorsal and ventral views. Scale bars: 12.0 mm.

Figures 73 [Fig F14] [Fig F15] 91

Euscorpiops validus  Di et al. 2010: 14–21, Figs 1–32, tabs. 1–2.

####### Type locality.

China, Yunnan Province, Mengzi District.

####### Type material examed.

Male holotype, China: Yunnan, Honghe Prefecture, 9/IX/2009, Junyun Huang leg (Ar.-MWHU-YNHH0901). Allotype female (Ar.-MWHU-YNHH0902); paratypes, 4 males, and 4 females (Ar.-MWHU-YNHH0903–06, Ar.-MWHU-YNHH0907–10), same data as holotype.

####### Diagnosis.

Medium-sized scorpions, total length 50.0–59.8 mm. It can be distinguished from other species of Euscorpiops by having thicker chelas. It can be ditinguished from other Euscopiops species from Yunnan by the following features: pedipalp patella with 9 to 10 (rarely 11 or 8) ventral trichobothria; chela strong, length/width ratio: 2.9–3.2 (mean 3.0 in 3 males, and 3.1 in 4 females); pectinal fulcra present (obsolete in some females); chela fingers obviously curved; pectinal teeth: 7–8; pectinal fulcra present and small. Euscorpiops validus can be ditinguished from related Euscopiops species by the following features: in Euscorpiops shidian there are 11 (rarely 10 or 12) ventral trichobothria on pedipalp patella, chela length/width ratio higher than 3.2 in Euscorpiops shidian, higher than 3.3 in Euscorpiops yangi and higher than 3.4 in Euscorpiops xui; chela fingers clearly curved in Euscorpiops validus, whereas in Euscorpiops shidian they are nearly straight, in Euscorpiops kubani female nearly straight and in Euscorpiops yangi they are slightly undulated in both sexes without sexual dimorphism; chela manus flat in Euscorpiops validus, whereas in Euscorpiops vachoni rounded.

####### Description.

See Di et al. (2010a).

**Figures 77–84. F14:**
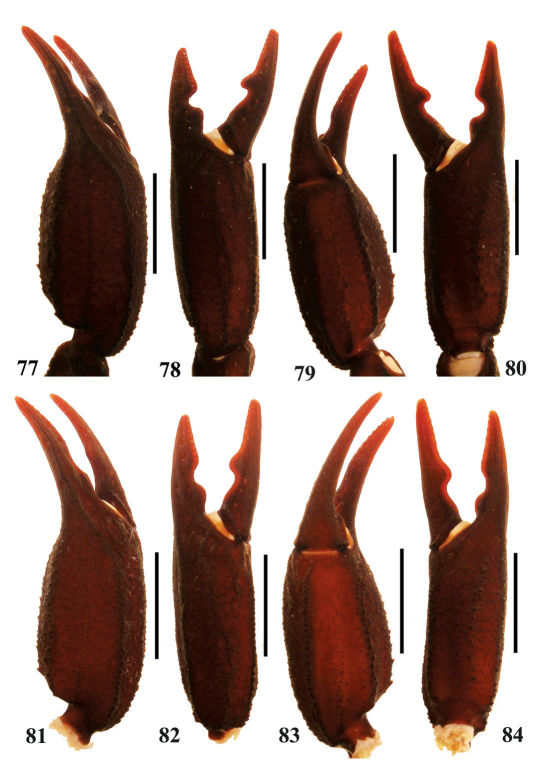
Euscorpiops validus. **77–80** Male holotype (Ar.-MWHU-YNHH0901). Chela dorsal, external, ventral and internal aspects **81–84** Female allotype (Ar.-MWHU-YNHH0902). Chela dorsal, external, ventral and internal aspects.Scale bars: 6.0 mm.

**Figures 85–90. F15:**
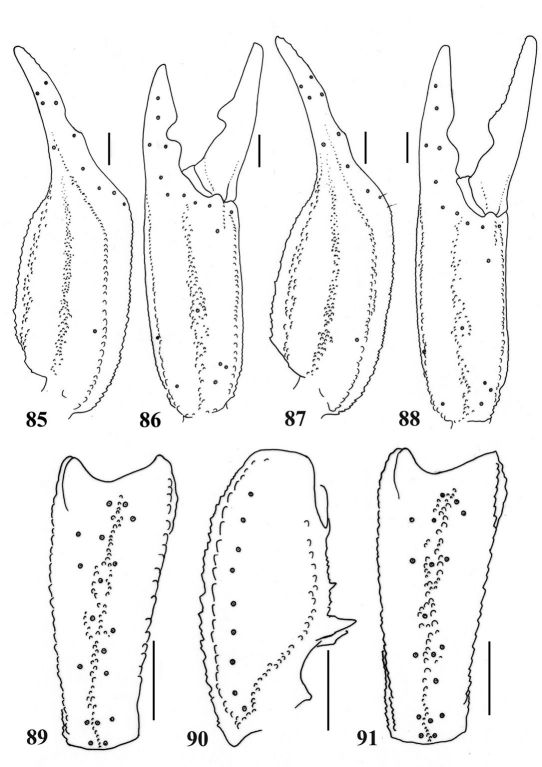
Euscorpiops validus. Male holotype (Ar.-MWHU-YNHH0901). **85–86** Chela dorsal and external aspects **89–90** Patella external and ventral aspects. Scale bars: 2.0 mm. **87–88, 91.** Euscorpiops validus. Female allotype (Ar.-MWHU-YNHH0902) **87–88** Chela dorsal and external aspects **91** Patella external aspect. Scale bars: 2.0 mm.

**Figures 92–95.**
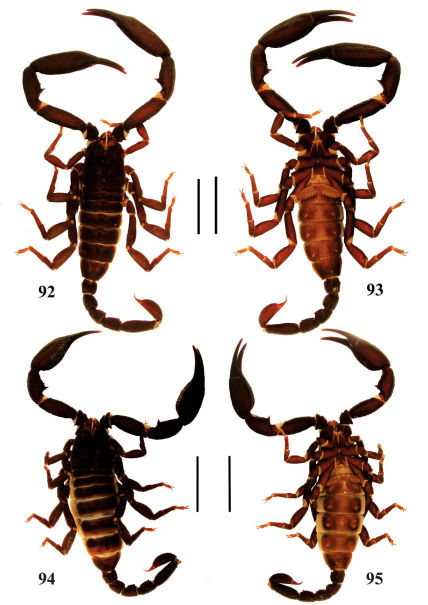
Habitus of Euscorpiops xui. **92–93** Male (Ar.-BHDC-YNML0901), dorsal and ventral views **94–95** Female (Ar.-BHDC-YNML0902), dorsal and ventral views. Scale bars: 12.0 mm.

**Figures 96–103. F17:**
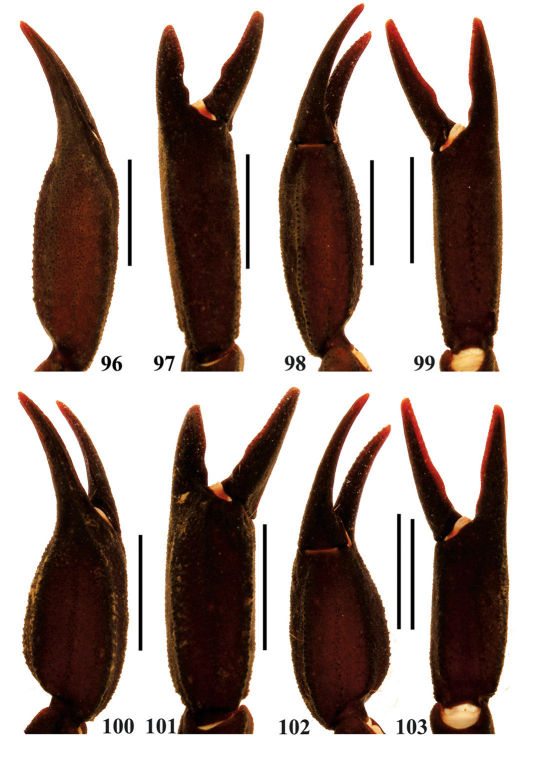
Euscorpiops xui. **96–99** Male (Ar.-BHDC-YNML0901). Chela dorsal, external, ventral and internal aspects **100–103** Female (Ar.-BHDC-YNML0902). Chela dorsal, external, ventral and internal aspects.Scale bars: 6.0 mm.

**Figures 104–110.**
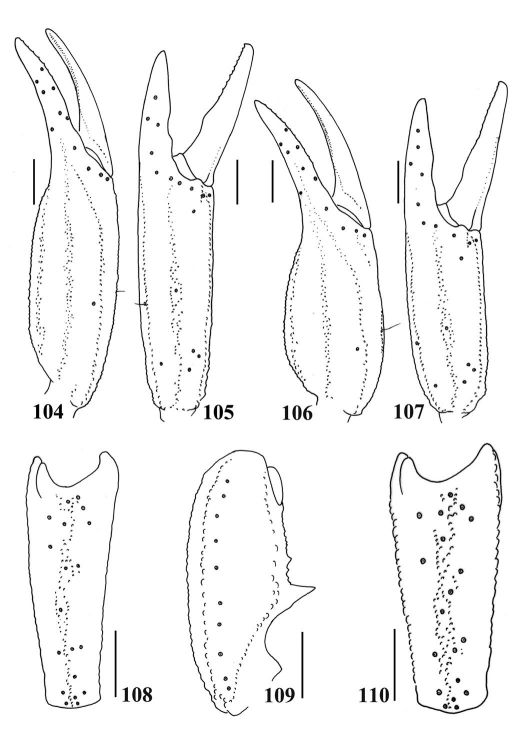
104–105, 108–109.Euscorpiops xui. Male (Ar.-BHDC-YNML0901) **104–105** Chela dorsal and external aspects **108–109** Patella external and ventral aspects. Scale bars: 2.0 mm. **106–107, 110.** Euscorpiops xui. Female (Ar.-BHDC-YNML0902) **106–107** Chela dorsal and external aspects **110** Patella external aspect. Scale bars: 2.0 mm.

####### Ecology.

This species is collected from moist mixed forest. They are found on the wall in the night and under stones in the day.

####### Distribution.

China (Yunnan, just the type locality).

###### Euscorpiops xui

Sun & Zhu, 2010

Figures 92 [Fig F17] 110

Euscorpiops xui Sun and Zhu 2010: 62–67, Figs 1–14, tab. 1.

####### Type locality.

China, Yunnan Province, Menglian District.

####### Type material.

Holotype female, China, Yunnan, Menglian District, Lafu Village, 22°08'N, 99°25'E, 15/VII/2009, Dr. Jishan Xu leg(MHBU); 1 female, 1 male, and 1 juvenile male paratypes, same data as holotype (MHBU).

####### Material examined.

Menglian County, 15/VII/2009, Lixiang Zhang leg, 1 male and 1 female. (BHDC).

####### Diagnosis

(Modified from Sun and Zhu 2010)**.** total length about 54.0–66.0 mm (2 males and 2 females); color dark brownish-red; chela, length/width ratio about 3.5 in females (3.4 and 3.6 in 2 records) and about 4.0 in males (4.0 and 4.1 in 2 records); dentate margin with a slight lobe on movable finger and corresponding notch on fixed finger in both males and females; patella with 19 or 18 external trichobothria (5 *eb*, 2 *esb*, 2 *em*, 4 *est*, 5 or 6 *et* , fig. 108; 6 *eb*, 2 *esb*, 2 *em*, 4 *est*, 5 *et*, Sun and Zhu 2010: fig. 3), and with 10 ventral trichobothria (4 specimens).

Euscorpiops xui appears to be closely related to Euscorpiops kubani, bothcan be distinguished by: male chela length/width ratio, about 4.0 in males and 3.5 in females, whereas it is about 3.1 in males and 2.9 in females in Euscorpiops kubani; pedipalp fingers nearly straight (Figs 105, 107), while in Euscorpiops kubani there is scalloped in males and nearly straight in females (Kovařík 2004; Sun and Zhu 2010).

Euscorpiops xui can be distinguished from other related species of the genus Euscorpiops by the following features: patella of pedipalp with 10 ventral trichobothria, whereas in Euscorpiops shidian with 11 (rarely 10 and 12 ); chela with a clear sexual dimorphism on length/width ratio: about 4.0 in males and 3.5 in females, compared with 2.7–3.2 in Euscorpiops kubani, 2.9–3.2 in Euscorpiops validus, and 2.6–2.8 in Euscorpiops puerensis; patella of pedipalp with 18–19 (rarely 18) external trichobothria in Euscorpiops xui, whereas 18 external trichobothria in Euscorpiops kubani, Euscorpiops shidian, Euscorpiops validus and Euscorpiops yangi; the coloration mainly dark brownish-red in Euscorpiops xui, but yellow brown in Euscorpiops vachoni.

####### Description.

See Sun and Zhu (2010).

####### Variation.

Measurements in table 1. Feature datasets in table 2.

####### Ecology.

This species is uncommon, collected from moist mixed forest close the villages.

####### Distribution.

China (Yunnan).

###### Euscorpiops yangi

Zhu, Zhang & Lourenço, 2007

Figures 111–117

Euscorpiops yangi Zhu et al. 2007: 20–25, Figs 1–22, tab. 1.

####### Type locality.

China, Yunnan Province, Maguan District.

####### Type material.

Male holotype, China, Yunnan Province, Maguan District, Gulingqing Town (23°00'N, 104°18'E), 20/VII/2006, Zizhong Yang and Yulong Wang leg, (Ar.-MHBU-0011); 3 males and 1 female paratypes, same data as holotype (1 male paratype in MNHN, the others in MHBU).

####### Diagnosis.

(Modified from Zhu et al. 2007)Medium-sized scorpion with total length 46.1 to 51.3 (4 males and 1 female); patella of pedipalp with 9 to 10 ventral trichobothria (Figs 115–117); chela narrow and elongated, the length/width ratio is 3.4 on males (4 specimens) and 3.3 on female (1 specimen), the chela length/carapace length ratio is equal or greater than 2.0; pedipalp fingers of males and females nearly straight (Figs 112, 114).

Euscorpiops yangi can be distinguished from other related species of the genus Euscorpiops by the following features: patella of pedipalp with 9 to 10 ventral trichobothria, whereas in Euscorpiops shidian with 11 (rarely 10 or 12); chela narrow and elongated; the length/ width ratio is 3.4 in males and 3.3 in females, compared with 2.7–3.2 in Euscorpiops kubani, 2.9–3.2 in Euscorpiops validus , and 2.6–2.8 in Euscorpiops puerensis. Euscorpiops yangi can be ditinguished from Euscorpiops xui by the following features: patella of pedipalp with 18 external trichobothria whereas in Euscorpiops xui with 18–19; chela with length/width ratio 3.4 in males and 3.3 in females, whereas on Euscorpiops xui withlength/width ratio 4.0–4.1 in males (2 specimens) and 3.4–3.6 in females (2 specimens).

####### Description.

See Zhu et al. (2007).

**Figures 111–117.**
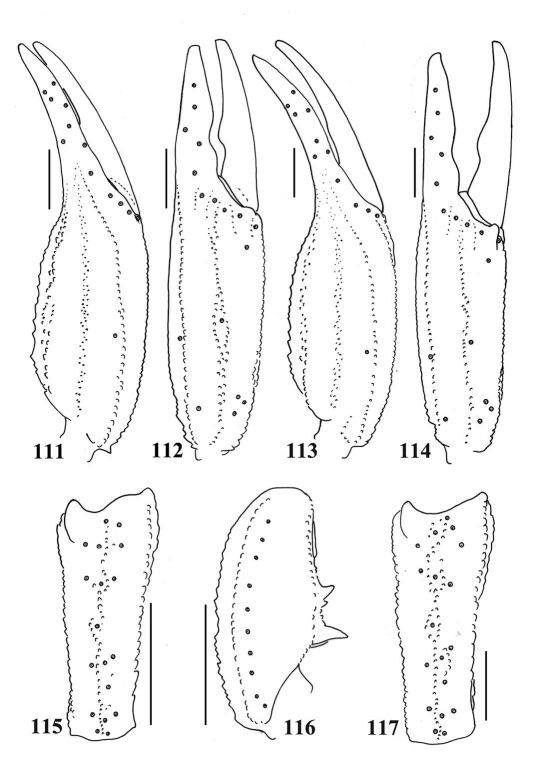
111–112, 115–116. (followed Zhu et al. 2007).Euscorpiops yangi. Male holotype (Ar.-MHU-0011). **111–112** Chela dorsal and external aspects **115–116** Patella external and ventral aspects. Scale bars: 2.0 mm. **113–114, 117.** (followed Zhu et al. 2007). Euscorpiops yangi, Female paratype **113–114** Chela dorsal and external aspects **117** Patella external aspect. Scale bars: 2.0 mm.

####### Ecology.

This species is uncommon, found under stones.

####### Distribution.

China (Yunnan).

##### Genus Scorpiops Peters, 1861

###### Scorpiops jendeki

Kovařík, 1994

Figures 118–122

Scorpiops hardwickii jendeki Kovařík 1994: 62, Figs 7–13, tab.1; Fet 2000: 492.
Scorpiops jendeki : Kovařík 2000: 180, 182, Figs 59–60, tabs. 1–3.

####### Type locality.

China, Yunnan, Gaoligongshan Nature Reserve 100 km west of Baoshan.

####### Type material.

Holotype female: China, Yunnan, Gaoligongshan Nature Reserve 100 km west of Baoshan; 1 female paratype (NMPC), 4 females paratypes (FKCP), 14–21/VI/1993, E. Jendek and O. Sausa leg.

####### Diagnosis.

(Taken from Kovařík 2000). Total length is 30–42.1 mm. Patella with 17 external trichobothria (5*eb*, 2 *esb*, 2 *em*, 4 *est*, 4 *et*) (Fig. 121) and 6–7 ventral trichobothria (6 specimens, Fig. 122). Pectinal teeth 4–5. Both males and females have fingers of pedipalps straight, without any flexure. The carapace bears very sparse large granules.

Scorpiops jendeki appears to be closely related to Scorpiops hardwickei (Gervais, 1843), both species have the same number of external and ventral trichobothria on the patella, and a similar length/width ratio of chela; however, in the latter the fingers of pedipalps are strongly flexed.

####### Description.

See (Kovařík (1994, 2000).

**Figures 118–122.**
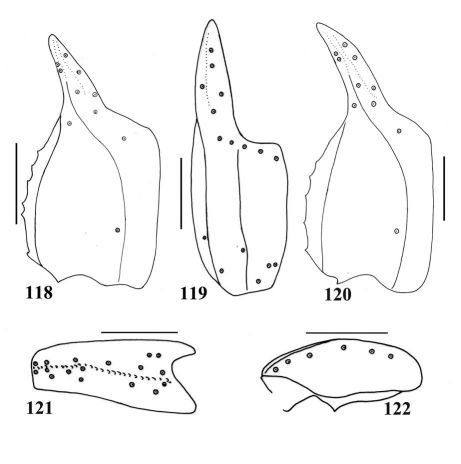
Scorpiops jendeki(followed Kovařík 1994, 2000).Male holotype. **118–119** Chela dorsal and external aspects **121–122** Patella external and ventral aspecta. Scale bars: 2.0 mm. **120** Scorpiops jendeki(followed Kovařík 2000).Female. Chela dorsal aspect. Scale bars: 2.0 mm.

####### Ecology.

This species is uncommon, collected from moist mixed forest and in the bark or leavers and moss.

####### Distribution.

China (Yunnan).

**Figure 123.**
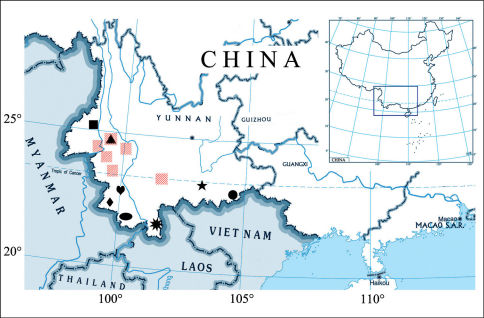
Map of China (Yunnan), showing the localities of the Scorpionesspecies. Map abbreviations: square, Scorpiops jendeki; ellipse, Euscorpiops kubani; heart, Euscorpiops puerensis; triangle, Euscorpiops shidian; polygon, Euscorpiops vachoni; pentagon, Euscorpiops validus; rhombus, Euscorpiops xui; circle, Euscorpiops yangi; red diagonal, Lychas mucronatus.

### Key to species of Scorpiones from Yunnan (China)

**Table:** 

1	Anterior margin of carapace retuse (Figs 1, 3), 5 pairs of lateral ocelli; telson with a subaculear tubercle	Lychas mucronatus (Fabricius)
–	Anterior margin of carapace deeply depressed (see Di et al. 2010b, Fig. 6), 3 pairs of lateral ocelli; telson without subaculear tubercle	2
2	Trichobothrium *Eb3* on the external surface of the chela located between trichobothria *Dt* and *Db*, pedipalp chela fingers straight; telson without annular ring	Scorpiops jendeki Kovarik
–	Trichobothrium *Eb3* on the external surface of the chela located between trichobothria *Dt* and *Est*; telson with an annular ring at the juncture of the vesicle with aculeus (Fig. 48)	3
3	Male pedipalp chela fingers strongly scalloped: with a pronounced lobe on the movable finger and a corresponding notch on fixed finger	4
–	Male pedipalp chela fingers slightly scalloped or straight: lobe and corresponding notch reduced or absent	7
4	Chela manus stout and rounded	Euscorpiops vachoni Qi, Zhu and Lourenco
–	Chela manus flattened dorsoventrally	5
5	Female pedipalp chela fingers nearly straight	Euscorpiops kubani Kovarik
–	Female pedipalp chela fingers scalloped	6
6	Chela length/width ratio: 2.9–3.2 (average 3.0 in males, 3.1 in females); pedipalp patella with 9 to 10 (rarely 11 or 8) ventral trichobothria; pectinal teeth 6–8 (rarely 8)	Euscorpiops validus Di, Cao, Wu and Li
–	Chela length/width ratio: 2.6–2.8 (average 2.7 on both sexes); pedipalp patella with 11 or 10 (rarely 10) ventral trichobothria; pectinal teeth count 7–8	Euscorpiops puerensis Di, Wu, Cao, Xiao and Li
7	Chela length/width ratio about 3.5 in females, about 4.0 in males; platella of pedipalp with 19 external trichobothria (rarely 18)	Euscorpiops xui Sun and Zhu
–	Chela length/width ratio 3.2–3.5 in both sexes; patella of pedipalp always with 18 external trichobothria	8
8	Number of trichobothria on ventral surface of patella: 11 (rarely 10 or 12), pedipalp chela fingers nearly straight (Figs 57, 61)	Euscorpiops shidian Qi, Zhu and Lourenco
–	Number of trichobothria on ventral surface of patella: 10 or 9, pedipalp chela fingers slightly undulated (Figs 112, 114)	Euscorpiops yangi Zhu, Zhang and Lourenco

## Supplementary Material

XML Treatment for Lychas mucronatus

XML Treatment for Euscorpiops kubani

XML Treatment for Euscorpiops puerensis

XML Treatment for Euscorpiops shidian

XML Treatment for Euscorpiops vachoni

XML Treatment for Euscorpiops validus

XML Treatment for Euscorpiops xui

XML Treatment for Euscorpiops yangi

XML Treatment for Scorpiops jendeki
